# Supplementary material for: An efficient Screening System in Yeast to Select a Hyperactive piggyBac Transposase for Mammalian Applications
Source: Int J Mol Sci. 2020 Apr 26;21(9):3064. doi: 10.3390/ijms21093064 (PMC7247424; doi:10.3390/ijms21093064)
Supplement: Supplementary file 1 [file ijms-21-03064-s001.pdf]

International Journal of *Molecular Sciences*

MDPI

Article

An efficient screening system in yeast to select a hyperactive *piggyBac* transposase for mammalian applications

Wen wen[1], Shanshan Song[1], Yuchun Han[1], Haibin Chen[1], Zhong Li, Xiangzhen Liu[1,*], Qijun Qian[1*]

1 Shanghai Cell Therapy Research Institute, Shanghai Cell Therapy Group, Shanghai 201805, China
* Correspondence: qian@shcell.org; Tel: +86-021-5959 3168

Supplementary Materials

Table S1. The sequence of transposases and promoter

Transposases / promoter	Nucleotide sequence
WT PBase	atgggtagttctttagacgatgagcatatcctctctgctcttctgcaaagcgatgacgagcttgttggtgaggattctgacag<br>tgaaatatcagatcacgtaagtgaagatgacgtccagagcgatacagaagaagcgtttatagatgaggtacatgaagtg<br>cagccaacgtcaagcggtagtgaaatattagacgaacaaaatgttattgaacaaccaggttcttcattggcttctaacaga<br>atcttgaccttgccacagaggactattagaggtaagaataaacattgttggtcaacttcaaagtccacgaggcgtagccga<br>gtctctgcactgaacattgtcagatctcaaagaggtccgacgcgtatgtgccgcaatatatatgacccacttttatgcttcaa<br>actattttttactgatgagataatttcggaaattgtaaaatggacaaatgctgagatatcattgaaacgtcgggaatctatga<br>caggtgctacatttcgtgacacgaatgaagatgaaatctatgctttctttggtattctggtaatgacagcagtgagaaaaga<br>taaccacatgtccacagatgacctctttgatcgatctttgtcaatggtgtacgtctctgtaatgagtcgtgatcgttttgattttt<br>gatacgatgtcttagaatggatgacaaaagtatacggcccacacttcgagaaaacgatgtatttactcctgttagaaaaat<br>atgggatctctttatccatcagtgcatacaaaattacactccaggggctcatttgaccatagatgaacagttacttggttttag<br>aggacggtgtccgtttaggatgtatatcccaaacaagccaagtaagtatggaataaaaatcctcatgatgtgtgacagtg<br>gtacgaagtatatgataaatggaatgccttatttgggaagaggaacacagaccaacggagtaccactcggtgaatactac<br>gtgaaggagttatcaaagcctgtgcacggtagttgtcgtaatattacgtgtgacaattggttcacctcaatccctttggcaaa<br>aaacttactacaagaaccgtataagttaaccattgtgggaaccgtgcgatcaaacaaacgcgagataccggaagtactga<br>aaaacagtcgctccaggccagtgggaacatcgatgttttgttttgacggaccccttactctcgtctcatataaaccgaagcc<br>agctaagatggtatacttattatcatcttgtgatgaggatgcttctatcaacgaaagtaccggtaaaccgcaaatggttatgt<br>attataatcaaactaaaggcggagtggacacgctagaccaaatgtgttctgtgatgacctgcagtaggaagacgaatag<br>gtggcctatggcattattgtacggaatgataaacattgcctgcataaattcttttattatatacagccataatgtcagtagcaa<br>gggagagaaggttcaaagtcgcaaaaaatttatgagaaacctttacatgagcctgacgtcatcgtttatgcgtaagcgttt<br>agaagctcctactttgaagagatatttgcgcgataatatctctaatattttgccaaatgaagtgcctggtacatcagatgaca<br>gtactgaagagccagtaatgaaaaaacgtacttactgtacttactgcccctctaaaataaggcgaaaggcaaatgcatcgt<br>gcaaaaaatgcaaaaaagttatttgtcgagagcataatattgatatgtgccaaagttgtttctgataa
Optimized hyPBase	atgggcagcagcctggacgacgagcacatcctgagcgccctgctgcagagcgacgacgagctggtgggcgaggaca<br>gcgacagcgaggtgagcgaccacgtgagcgaggacgacgtgcagagcgacaccgaggaggccttcatcgacgaggt<br>gcacgaggtgcagcccaccagcagcggcagcgagatcctggacgagcagaacgtgatcgagcagcccggcagcagc<br>ctggccagcaaccgcatcctgaccctgccccagcgcaccatccgcggcaagaacaagcactgctggagcaccagcaag<br>cccacccgccgcagccgcgtgagcgccctgaacatcgtgcgcagccagcgcggccccacccgcatgtgccgcaacatct<br>acgacccccctgctgtgcttcaagctgttcttcaccgacgagatcatcagcgagatcgtgaagtggaccaacgccgagatca<br>gcctgaagcgccgcgagagcatgaccagcgccaccttccgcgacaccaacgaggacgagatctacgccttcttcggcat<br>cctggtgatgaccgccgtgcgcaaggacaaccacatgagcaccgacgacctgttcgaccgcagcctgagcatggtgtac<br>gtgagcgtgatgagccgcgaccgcttcgacttcctgatccgctgcctgcgcatggacgacaagagcatccgccccaccct<br>gcgcgagaacgacgtgttcacccccgtgcgcaagatctgggacctgttcatccaccagtgcatccagaactacaccccccg

	gcgcccacctgaccatcgacgagcagctgctgggcttccgcggccgctgccccttccgcgtgtacatccccaacaagccc<br>agcaagtacggcatcaagatcctgatgatgtgcgacagcggcaccaagtacatgatcaacggcatgccctacctgggcc<br>gcggcacccagaccaacggcgtgcccctgggcgagtactacgtgaaggagctgagcaagcccgtgcacggcagctgc<br>cgcaacatcacctgcgacaactggttcaccagcatcccccctggccaagaacctgctgcaggagccctacaagctgaccat<br>cgtgggcaccgtgcgcagcaacaagcgcgagatccccgaggtgctgaagaacagccgcagccgccccgtgggcacca<br>gcatgttctgcttcgacggcccccctgaccctggtgagctacaagcccaagcccgccaagatggtgtacctgctgagcagct<br>gcgacgaggacgccagcatcaacgagagcaccggcaagccccagatggtgatgtactacaaccagaccaagggcgg<br>cgtggacaccctggaccagatgtgcagcgtgatgacctgcagccgcaagaccaaccgctggcccatggccctgctgtac<br>ggcatgatcaacatcgcctgcatcaacagcttcatcatctacagccacaacgtgagcagcaagggcgagaaggtgcaga<br>gccgcaagaagttcatgcgcaacctgtacatgggcctgaccagcagcttcatgcgcaagcgcctggaggcccccaccct<br>gaagcgctacctgcgcgacaacatcagcaacatcctgcccaaggaggtgcccggcaccagcgacgacagcaccgagg<br>agcccgtgatgaagaagcgcacctactgcacctactgccccagcaagatccgccgcaaggccagcgccagctgcaaga<br>agtgcaagaaggtgatctgccgcgagcacaacatcgacatgtgccagagctgcttctaa
Optimized WT PBase	atgggcagcagcctggacgacgagcacatcctgagcgccctgctgcagagcgacgacgagctggtgggcgaggaca<br>gcgacagcgagatcagcgaccacgtgagcgaggacgacgtgcagagcgacaccgaggaggccttcatcgacgaggt<br>gcacgaggtgcagcccaccagcagcggcagcgagatcctggacgagcagaacgtgatcgagcagcccggcagcagc<br>ctggccagcaaccgcatcctgaccctgccccagcgcaccatccgcggcaagaacaagcactgctggagcaccagcaag<br>agcacccgccgcagccgcgtgagcgccctgaacatcgtgcgcagccagcgcggccccacccgcatgtgccgcaacatct<br>acgacccccctgctgtgcttcaagctgttcttcaccgacgagatcatcagcgagatcgtgaagtggaccaacgccgagatca<br>gcctgaagcgccgcgagagcatgaccggcgccaccttccgcgacaccaacgaggacgagatctacgccttcttcggcat<br>cctggtgatgaccgccgtgcgcaaggacaaccacatgagcaccgacgacctgttcgaccgcagcctgagcatggtgtac<br>gtgagcgtgatgagccgcgaccgcttcgacttcctgatccgctgcctgcgcatggacgacaagagcatccgccccaccct<br>gcgcgagaacgacgtgttcacccccgtgcgcaagatctgggacctgttcatccaccagtgcatccagaactacaccccccg<br>gcgcccacctgaccatcgacgagcagctgctgggcttccgcggccgctgccccttccgcatgtacatccccaacaagccc<br>agcaagtacggcatcaagatcctgatgatgtgcgacagcggcaccaagtacatgatcaacggcatgccctacctgggcc<br>gcggcacccagaccaacggcgtgcccctgggcgagtactacgtgaaggagctgagcaagcccgtgcacggcagctgc<br>cgcaacatcacctgcgacaactggttcaccagcatcccccctggccaagaacctgctgcaggagccctacaagctgaccat<br>cgtgggcaccgtgcgcagcaacaagcgcgagatccccgaggtgctgaagaacagccgcagccgccccgtgggcacca<br>gcatgttctgcttcgacggcccccctgaccctggtgagctacaagcccaagcccgccaagatggtgtacctgctgagcagct<br>gcgacgaggacgccagcatcaacgagagcaccggcaagccccagatggtgatgtactacaaccagaccaagggcgg<br>cgtggacaccctggaccagatgtgcagcgtgatgacctgcagccgcaagaccaaccgctggcccatggccctgctgtac<br>ggcatgatcaacatcgcctgcatcaacagcttcatcatctacagccacaacgtgagcagcaagggcgagaaggtgcaga<br>gccgcaagaagttcatgcgcaacctgtacatgagcctgaccagcagcttcatgcgcaagcgcctggaggcccccaccct<br>gaagcgctacctgcgcgacaacatcagcaacatcctgcccaacgaggtgcccggcaccagcgacgacagcaccgagg<br>agcccgtgatgaagaagcgcacctactgcacctactgccccagcaagatccgccgcaaggccaacgccagctgcaaga<br>agtgcaagaaggtgatctgccgcgagcacaacatcgacatgtgccagagctgcttctaa
GALS promoter	ggaagactctcctccgtgcgtcctcgtcttcaccggtcgcgttcctgaaacgcagatgtgcctcgcgccgcactgctccgaa<br>caataaagattctacaatactagcttttatggttatgaagaggaaaaattggcagtaacctggccccacaaaccttcaaatg<br>aacgaatcaaattaacaaccataggatgataatgcgattagtttttttagccttatttctggggtaattaatcagcgaagcgat<br>gatttttgatctattaacagatatataaatgcaaaaactgcataaccactttaactaatactttcaacattttcggtttgtattact<br>tcttattcaaatgtaataaaagtatcaacaaaaaattgttaatatacctctatactttaacgtcaaggagaaaaaaccccgga<br>t

Table S2. The mutations of candidates

Candidates	Relative activity in yeast	Relative activity in CHO cells	Mutations
Optimized hyPBase	1	1	
h31-11	9.7	1.19	186, atc→gtc, I186V
h31-15	10.7	0.66	438, gag→ggg, E438G 458, acc→tcc, T458S 562, gtg→gag, V562E
h31-27	13.6	0.45	4, gct→gtt, A4V 30, gag→ggg, E30G 44, gtg→gcg, V44A<br>68, agc→ggc, S68G 105, aag→agg, K105R 122, ctg→ccg, L122P<br>198, gtg→atg, V198M 392, ctg→cta, L392L 567, acc→gcc, T567A
h31-30	14.3	0.73	113, ccc→ctc, P113L 126, cgc→cac, R126H 313, acc→tcc, T313S
h32-20	11.9	0.39	91, cgc→tgc, R91C 296, aac→aaa, N296K 593, cgc→cgt, R593R
h33-14	8	1.23	394, aac→acc, N394T 540, gac→gat, D540D
h33-23	11.7	0.53	267, cag→caa, Q267Q 404, atg→acg, I404T 430, tgc→cgc, C430R<br>602, agc→tgc, S602C 575, aag→gag, K575E
h34-7	3	0.77	59, gac→ggc, D59G 423, atg→acg, M423T 461, cag→caa, Q461Q
h34-22	14.6	0.85	71, agc→ggc, S71G 80, atc→gtc, I80V 593, cgc→cgt, R593R
h61-4	9.8	0.93	144, tgc→agc, C144S
h61-5	7	1.08	156, gag→gtg, E156V 467, acc→tcc, T467S 507, cag→cgg, Q507R
h61-14	8.2	1	551, ccc→ctc, P551L
h62-9	2.48	0.58	70, ggc→gac, G70D 489, atc→acc, I489T
h62-14	2.67	0.6	36, agc→ggc, S36G 367, aac→agc, N367S 518, atg→aag, M518K<br>571, acc→gcc, T571A
h62-16	2.88	1.62	92, atc→aac, I92N 119, gtg→gcg, V19A 300, aag→aaa, K300K<br>601, cag→cgg, Q601R

Figure S1

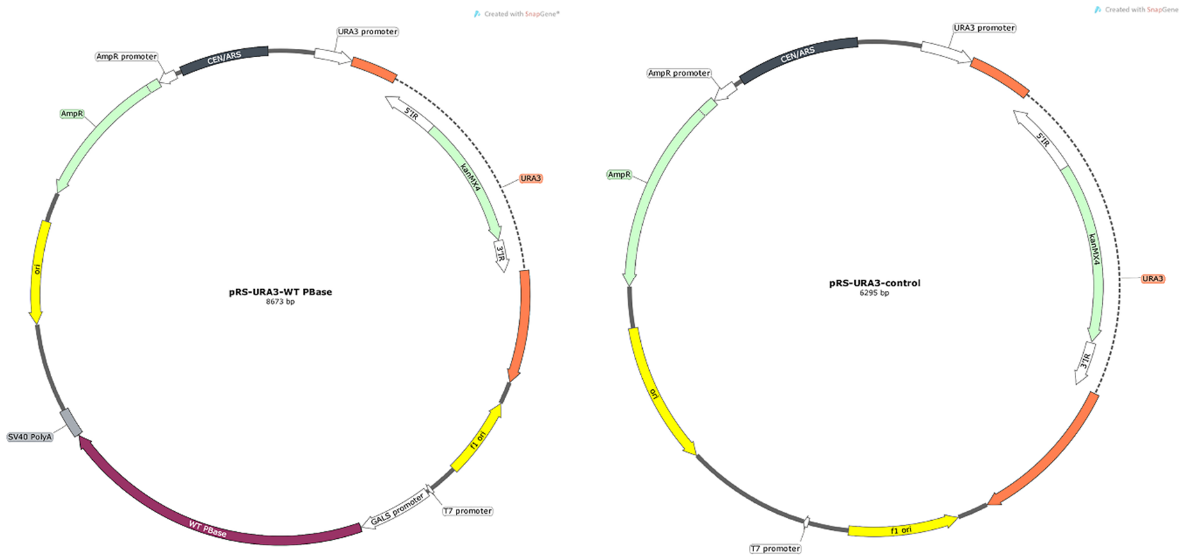

Figure S1. The map of pRS-URA3-WT PBase and pRS-URA3-control. The *ura3* gene was separated by the *piggyBac* transposon at TTAA site, which completely disturbed the normal expression. The *piggyBac* transposase with galactose-inducible GALS promoter was inserted into the multiple cloning site.

Figure S2

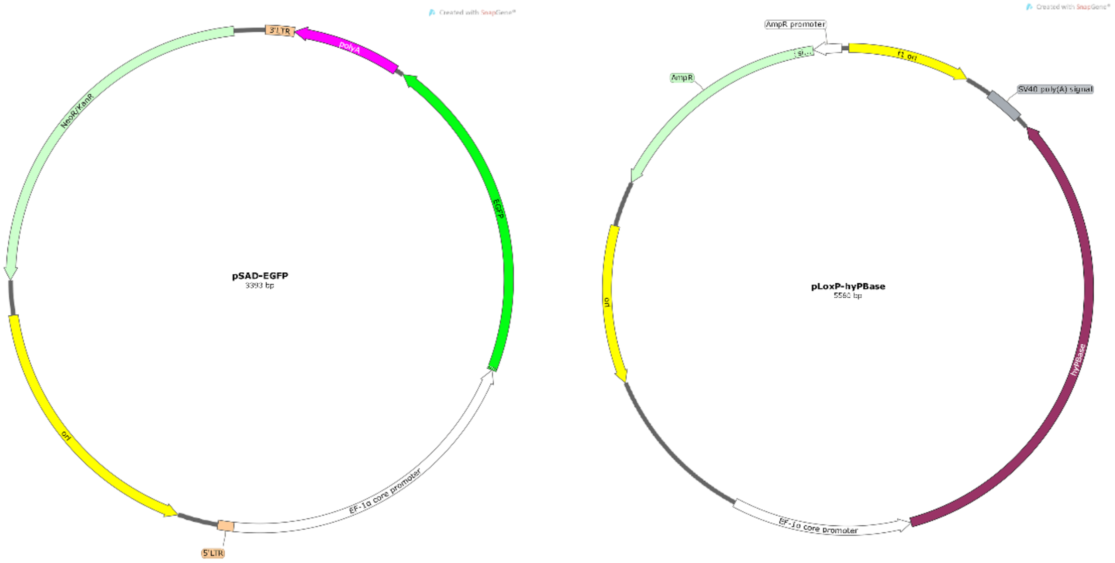

Figure S2. The map of the two-plasmid transposon system in mammalian cells. The transposon plasmid pSAD-EGFP contains the *egfp* gene that can express green fluorescent protein between 5′ LTR and 3′ LTR. The *piggBac* transposase can recognize and combine to the 5′ LTR and 3′ LTR areas, and then removed the transposon. The transposase plasmid contains the transposase optimized hyPBase or its mutants under the EF-1α core promoter.

Figure S3

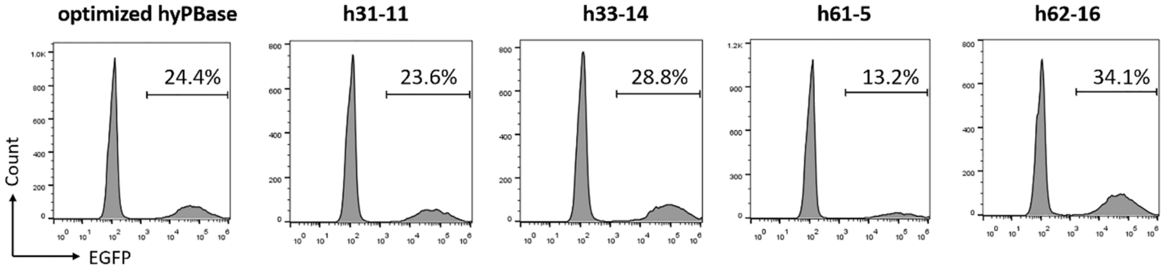

Figure S3. Relative activities of 4 hyperactive *piggyBac* transposase mutants in T Cells. The two-plasmid transposon system (pLoxP-optimized hyPBase/ pLoxP-mutant to express transposase, pSAD-EGFP to express transposon) were electrotransformed into human T cells. After 10 days culture, positive cells which completed gene transposition were measured by flow cytometry. The X-axis represented the intensity of EGFP, and the Y-axis represented the number of cells. The values represented the percent of EGFP cells in each sample.

Figure S4

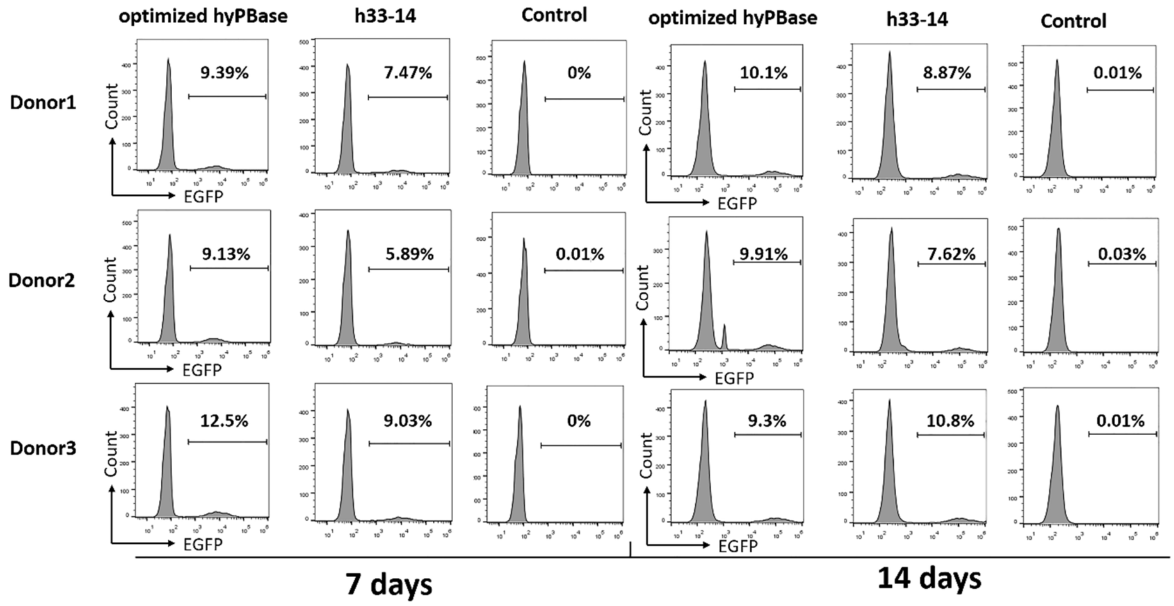

Figure S4. Relative activities of h33-14 in T Cells. The two-plasmid transposon system (pLoxP-optimized hyPBase/ pLoxP- h33-14 to express transposase, pSAD-EGFP to express transposon) were electrotransformed into human T cells. After 7 days and 14 days culture, positive cells which completed gene transposition were measured by flow cytometry. The right were the control cells that transformed into the transposon plasmid pSAD-EGFP only. The X-axis represented the intensity of EGFP, and the Y-axis represented the number of cells. The values represented the percent of EGFP cells in each sample. The experiment was repeated by three groups T cells from 3 different donors.

Figure S5

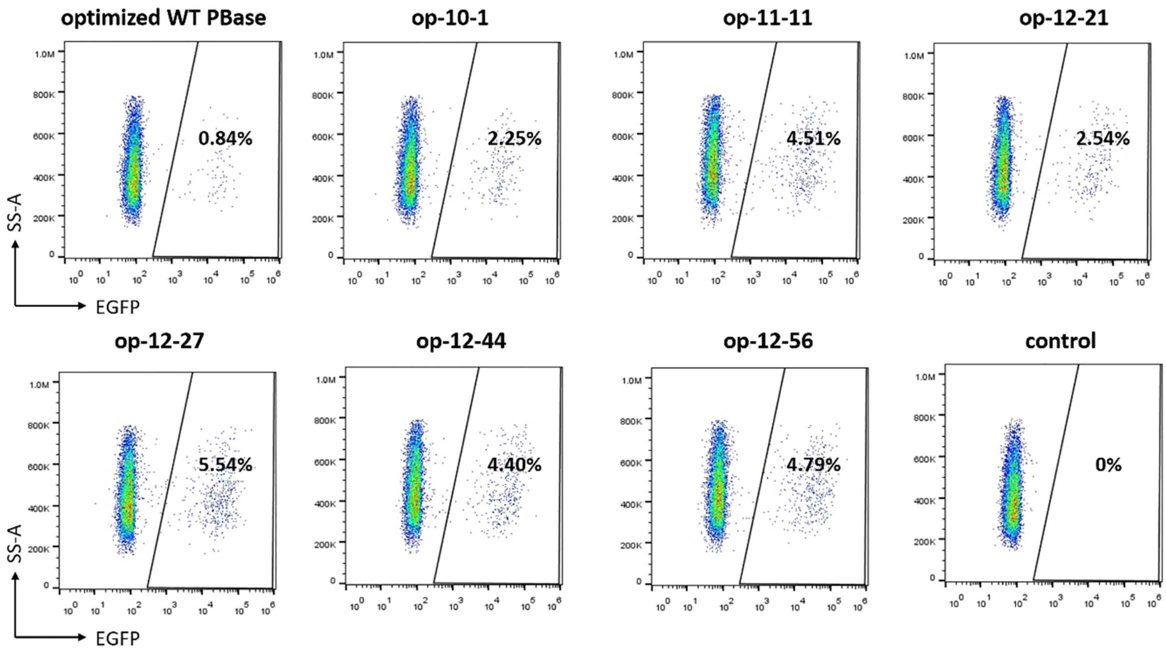

Figure S5. Relative activities of 6 mutants of optimized WT PBase in T Cells. The two-plasmid transposon system (pLoxP-optimized WT PBase/ pLoxP-mutant to express transposase, pSAD-EGFP to express transposon) were electrotransformed into human T cells. After 10 days culture, positive cells which completed gene transposition were measured by flow cytometry. The last was the control cells that transformed into the transposon plasmid

pSAD-EGFP only. The X-axis represented the intensity of EGFP, and the Y-axis represented the SS-A of cells. The values represented the percent of EGFP cells in each sample.

Figure S6

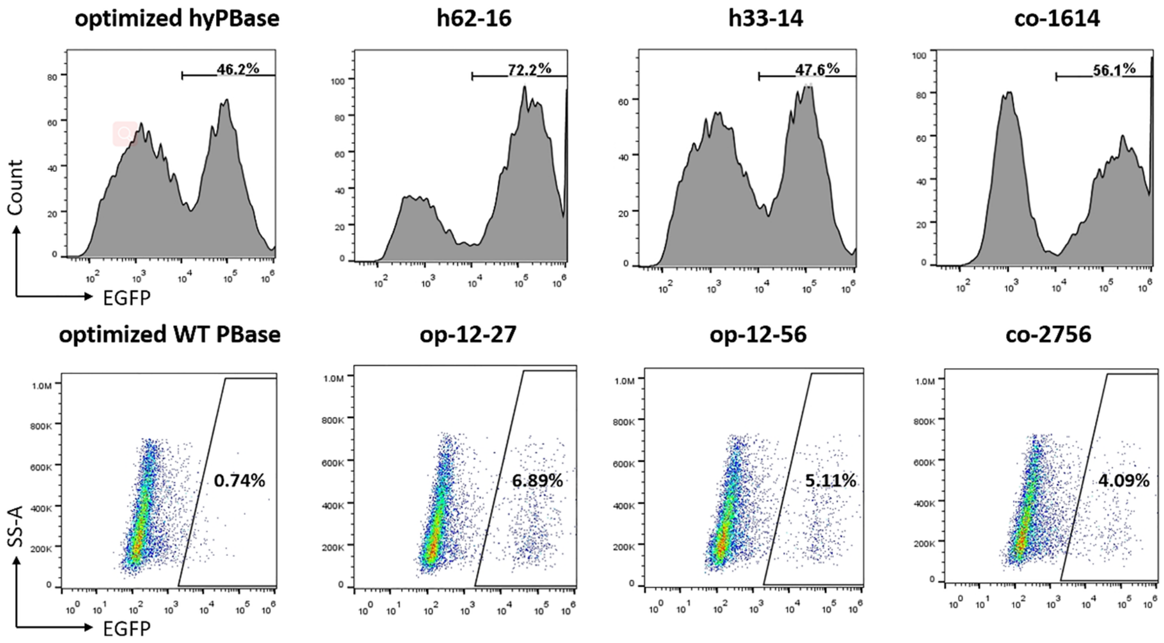

Figure S6. Relative activities of the optimized hyPBase-combined mutant (upper) and the optimized WT PBase-combined mutant (lower) in CHO Cells. The two-plasmid transposon system were electrotransformed into CHO cells. After 14 days culture, positive cells which completed gene transposition were measured by flow cytometry. The mutant co-1614 is the mutant that integrated the mutations of h33-14 and h62-16 into one sequence. The mutant co-2756 is the mutant that integrated the mutations of op12-27 and op12-56 into one sequence. The X-axis represented the intensity of EGFP, and the Y-axis represented the number of cells (upper) or the SS-A of cells (lower). The values represented the percent of EGFP cells in each sample.
